# Non-Contact Inspection of Railhead via Laser-Generated Rayleigh Waves and an Enhanced Matching Pursuit to Assist Detection of Surface and Subsurface Defects

**DOI:** 10.3390/s21092994

**Published:** 2021-04-24

**Authors:** Imran Ghafoor, Peter W. Tse, Javad Rostami, Kim-Ming Ng

**Affiliations:** Croucher Optical Nondestructive Testing Laboratory, Department of System Engineering and Engineering Management, City University of Hong Kong, Tat Chee Avenue, Kowloon, Hong Kong, China; Ighafoor3-c@my.cityu.edu.hk (I.G.); jrostami2-c@my.cityu.edu.hk (J.R.); kimmingng2-c@my.cityu.edu.hk (K.-M.N.)

**Keywords:** laser inspection system, rayleigh wave, finite element simulation, matching pursuit, rail track, non-destructive testing

## Abstract

Laser ultrasonic technology can provide a non-contact, reliable and efficient inspection of train rails. However, the laser-generated signals measured at the railhead are usually contaminated with a high level of noise and unwanted wave components that complicate the identification of defect echoes in the signal. This study explores the possibility of combining laser ultrasonic technology (LUT) and an enhanced matching pursuit (MP) to achieve a fully non-contact inspection of the rail track. A completely non-contact laser-based inspection system was used to generate and sense Rayleigh waves to detect artificial surface horizontal, surface edge, subsurface horizontal and subsurface vertical defects created at railheads of different dimensions. MP was enhanced by developing two novel dictionaries, which include a finite element method (FEM) simulation dictionary and an experimental dictionary. The enhanced MP was used to analyze the experimentally obtained laser-generated Rayleigh wave signals. The results show that the enhanced MP is highly effective in detecting defects by suppressing noise, and, further, it could also overcome the deficiency in the low repeatability of the laser-generated signals. The comparative analysis of MP with both the FEM simulation and experimental dictionaries shows that the enhanced MP with the FEM simulation dictionary is highly efficient in both noise removal and defect detection from the experimental signals captured by a laser-generated ultrasonic inspection system. The major novelty contributed by this research work is the enhanced MP method with the developments of, first, an FEM simulation dictionary and, second, an experimental dictionary that is especially suited for Rayleigh wave signals. Third, the enhanced MP dictionaries are created to process the Rayleigh wave signals generated by laser excitation and received using a 3D laser scanner. Fourth, we introduce a pioneer application of such laser-generated Rayleigh waves for inspecting surface and subsurface detects occurring in train rails.

## 1. Introduction

In the railroad industry, infrastructure safety is its top priority. However, maintenance of the rail track structure has been one of the most significant challenges since railroading began. Rail defects may present the primary source of irregularities, which not only threatens the safety of the vehicle [1] but also affects the interaction with the overhead contact line [2]. Among the major flaws found in rail tracks are surface and subsurface defects. If they are not detected effectively, they may increase in size speedily and become a threat to the rail track’s integrity. Therefore, the detection of these rail defects is a serious challenge for the rail management community. Generally, non-destructive testing (NDT) is used for rail track inspection. Many types of NDTs are available for the detection of flaws in the rail track. These include visual inspection, eddy current testing [3], ultrasonic testing [4,5,6,7] and the acoustic emission (AE) method [8].

The laser ultrasonic technique (LUT) is one of the latest candidates for non-destructive testing. Several research groups have investigated LUT for the inspection of rail tracks [9,10,11,12,13,14,15]. However, in these works, for the non-contact detection of laser-generated ultrasonic waves, mostly EMAT [9] and air-coupled transducers [11,12] were used. One of these transducer’s major drawbacks is the limited lift-off distance because the signal amplitude and signal-to-noise ratio (SNR) significantly reduce as the distance between the sample and transducer increases. Use of the laser generation–laser receiving technique provides another advanced non-contact method. A scanning laser vibrometer provides the possibility of measuring the responses at a large number of measurement points. Further, this laser ultrasonic technique can produce wavefield images of much higher spatial resolution than conventional transducers [16,17]. Although this laser generation–laser receiving technique has been used for defect detection in different geometries [18,19,20,21,22,23,24,25,26,27], minimal research is available for rails [13,28]. Therefore, there is a great need to conduct more research to investigate a fully non-contact laser-based inspection system that can detect the rail’s surface and subsurface defects. However, the signals generated through a non-contact inspection system usually have a lower signal-to-noise ratio compared to that of contact-based methods. This is mainly because of the difference in magnitudes of stresses exerted by these excitation methods to generate elastic waves [29]. The laser excitation in a non-contact inspection system usually has lower excitation energy and hence the generated ultrasonic signals have a poor SNR. Thus, the laser-generated ultrasonic signals generally contain a significant noise level compared to the propagating waves. Consequently, identifying defect-related information from such a signal is very difficult, and the implementation of an appropriate signal processing technique is inevitable. Another limitation of laser excitation is that the generated time-domain signals are usually non-repeatable, i.e., when the measurements are repeated at the same sensing location, the shape of the incident wave packet and noise level slightly changes.

Matching pursuit (MP) is a robust algorithm that is used to analyze stationary and non-stationary ultrasonic signals. It relies on an adoptive decomposition of a given signal into weighted linear combinations of basis functions known as atoms from a pre-defined extensive and redundant dictionary of functions [30]. Several research groups [31,32,33] have used matching pursuit-based algorithms to extract useful features from guided wave signals generated on structures as simple as plates [34] and pipes [31]. However, as of today, MP’s capabilities have not been investigated to extract desired features from a Rayleigh wave signal recorded at a complex geometry such as a rail. Further, in previous studies, matching pursuit was mainly used to extract desired features and de-noise ultrasonic signals excited through contact-based methods such as the comb PVDF transducer [34] and magnetostrictive transducer [31,33]. However, MP’s capability to de-noise laser-generated ultrasonic signals has not been investigated thus far. Therefore, the current research focus is defect detection and noise removal from laser-generated Rayleigh wave signals recorded at railheads. By designing an appropriate dictionary, MP can be highly efficient in extracting useful features from a laser-generated signal by de-noising it. In the literature, dictionary designs for ultrasonic waves are mostly based on the Gabor model [34], Gaussian model [35] and tone burst function [32]. However, for effective approximations, these synthetic dictionaries were usually produced very large in size, which costs a large computation time for the approximation of one echo.

A dictionary consisting of experimental signals can provide an excellent match between its atom and a given laser-generated ultrasonic signal. Therefore, there is no need to keep an abundant number of atoms in the dictionary as desired results can be achieved by keeping the size of the dictionary limited. Further, an experimental dictionary designed for a specific structure can also ensure the reliability of a similar simulation dictionary by comparing their results. Once created, an experimental dictionary for a specific geometry such as a railhead could be applicable to railheads of all sizes. However, as per an extensive state-of-the-art literature review, an experimental dictionary consisting of laser-generated ultrasonic signals has not been developed. Therefore, this research’s major novelty is the enhancement of matching pursuit by developing an experimental dictionary consisting of laser-generated Rayleigh wave signals recoded at railheads. An utterly non-contact laser-based inspection system was used for railhead defect detection and to design the experimental dictionary. The laser-generated ultrasonic waves were made narrowband with the help of the authors’ [36] newly designed optical system called the Sagnac interferometer-based optical system (SIOS), which produces the irradiation of a pulsed laser as a line array pattern (LAP) by using several optical lenses.

An FEM can also model realistic laser-generated Rayleigh waves and provides flexibility in controlling the parameters (for example, frequency, numbers of cycles, amplitude and phase) of the signal. Further, by using commercially available FEM software, the computational time for dictionary design can be saved too [37]. The benefits of using an FEM simulation dictionary consisting of laser-generated Rayleigh waves have also not been investigated thus far. Therefore, the development of an FEM simulation dictionary composed of simulated laser-generated Rayleigh signals is another novelty of this study. The FEM simulation dictionary was designed by developing a 2D finite element simulation model for the thermoelastic generation of narrowband Rayleigh waves in railheads. The functioning of the enhanced MP with experimental and FEM simulation dictionaries was tested successfully on laser-generated Rayleigh wave signals recorded at railheads of different dimensions having different types of surface and subsurface defects. Further, a comparison analysis of the enhanced MP with experimental and FEM simulation dictionaries in terms of removing noise and defect detection from laser-generated signals is also presented.

This paper’s structure is as follows: After describing the theory of matching pursuit in Section 2, experimental dictionary design, along with details on the experimental arrangement and test specimens, is provided in Section 3. Then, FEM simulation dictionary design and a detailed explanation of the FEM simulation modal are presented in Section 4. The results and discussion are presented separately in Section 5, and the conclusion of the study is given at the end.

## 2. Matching Pursuit

The matching pursuit (MP) algorithm is a greedy algorithm that helps to approximate a given signal *x*(*t*) by iteratively selecting the atom *a*(*t*) having the best match with the original signal from a redundant dictionary D [30,31]. The dictionary is composed of a matrix of parameterized waveform atoms in columns. The atoms’ sampling frequency is the same as the signal to be approximated *x*(*t*). The waveform atom is usually presented by amplitude, phase, frequency or other vital parameters. The dictionary atoms are designed in a way that they match maximally with the significant wave packets and mismatch with unwanted components or noise.

In order to approximate the signal structures, the selection of components from the dictionary should be such that any required information enclosed in the signal could be reconstructed. During the iteration, when an atom produces a maximum inner product with the signal, the match is said to be the best. Mathematically, after *n* iterations from a redundant dictionary *D* = {*d_1_, d_2_, d_3_, …, d_m_*}, MP represents a signal as a linear combination of some best-matched atoms *f_n_*(*t*) plus a residue term *R_n_*(*t*) as follows:

(1) $$x\left(t\right)=f_{n}\left(t\right)+R_{n}\left(t\right)\;=\sum_{i=1}^{n}c_{i}a_{i}\left(t\right)+R_{n}\left(t\right),\;\;\;\;\;\;║a_{i}\left(t\right)║=1$$

where the real number {*c*_1_, *c*_2_,…, *c*_n_}∁R_n_ is the corresponding coefficient of the atom {*a*_1_, *a*_2_, *a*_3_,…, *a*_n_}∁ D} and determines its amplitude. The MP algorithm works in a way that it selects the basis and the corresponding coefficient iteratively. Additionally, for an optimum approximation, this iteration process continues until the second-order norm of the residual component becomes minimum.

(2) $$║R_{n}\left(t\right){║}_{2}=║\;x\left(t\right)-f_{n}\left(t\right){║}_{2}<\;\varepsilon$$

Here, *ε* is a constant, and its value depends on the signal’s noise level. Typically, the value of ε is undefined, which is set via a trial and error method [30]. The first step of MP is selecting a waveform atom a_0_ that best matches the given signal by evaluating the similarity by the inner product.

Let us assume that *n* − 1 ≥ 0 atoms are approximating the signal; then, for further calculations for *n* atoms, the MP algorithm is given as follows:

1. The inner product between the atoms and the residue *R_n_*_−1_(*t*) should be calculated from the dictionary, except those which were previously taken in the last iteration.
2. Now, from the dictionary *D*, produce the maximum inner product with *R_n_*_−1_(*t*) by utilizing an atom a:
  (3) $$\left|\left\langle R_{n-1},\;a_{n}\right\rangle \right|\geq \rho sup\left|\left\langle R_{n-1},a\right\rangle \right|,a\in D$$
  where 0 < σ ≤ 1 is a numeric value that does not depend on *n*.
3. The new residue can be calculated as
  (4) $$R_{n\;}\left(t\right)=R_{n-1}\left(t\right)-\langle R_{n-1}\left(t\right),a_{n}\left(t\right)\rangle a_{n}\left(t\right)$$
4. The signal’s *x*(*t*) new approximation with *n* atoms after *n*−1 decomposition interactions can be represented as
  (5) $$x\left(t\right)=\overset{n}{\underset{i=1}{\sum }}\left\langle x\left(t\right),a_{i}\left(t\right)\right\rangle a_{i}\left(t\right)+R_{n}\left(t\right)$$

### Dictionary Design Requirements

MP decomposition provides an extremely flexible signal representation because a wide variety of dictionaries can be used. The development of a suitable dictionary is very important in obtaining a useful decomposition and approximation of a laser-generated ultrasonic signal with MP. The dictionary’s design should be based on the previously available information (for example, number of cycles, time span, frequency, amplitude and phase) of the laser-generated Rayleigh wave propagation in a specific structure. Since the convergence of residual ‖*R*(*t*)‖^2^ does not depend on the type of elementary atom used for MP, there is an open choice to use any function to match the given signal. In this way, an optimal approximation could be achieved for a specific application. Further, two important properties of the dictionary: over-completeness and redundancy, should be considered. A dictionary is said to be over-complete when its number of atoms exceeds the length of the original signal, while redundancy indicates that orthogonality is not necessarily satisfied by the atoms in a dictionary. These properties help the waveform matching between the atoms and the signal to a maximum extent.

## 3. Development of Experimental Dictionary

**Figure 1:**
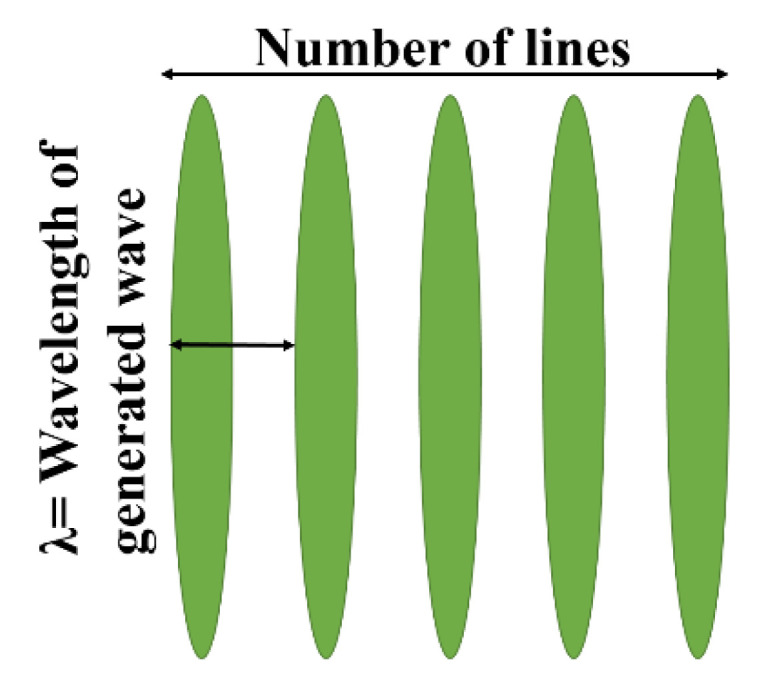
Schematic diagram of an LAP by using the optical system.

To design a dictionary consisting of experimental laser-generated signals, a completely non-contact laser system was used. This system is capable of producing and detecting the Rayleigh waves remotely by using excitation and detection laser sources. The ultrasonic waves generated by laser excitation are broad in bandwidth. However, in practical applications such as non-destructive testing, narrowband ultrasonic waves are usually preferred [18,38]. The laser-generated ultrasound can be made narrowband using spatial illumination in the form of a line array pattern [38,39]. A schematic illustration of a line array pattern (LAP) is given in Figure 1.

When a material is illuminated with a line array laser pattern, part of the optical energy will be absorbed by the area under irradiation. The characteristics of the generated ultrasonic waves are determined by the temporal and spatial distributions of the laser beam profile. Since the line spacing of an LAP governs the wavelength of the generated ultrasonic waves, by adjusting the LAP’s line spacing, ultrasonic waves of the desired frequency can be generated. To generate narrowband guided waves, the authors used a newly designed optical system called the integrated Sagnac interferometer-based optical system (SIOS) [36].

### 3.1. Specimen

**Figure 2:**
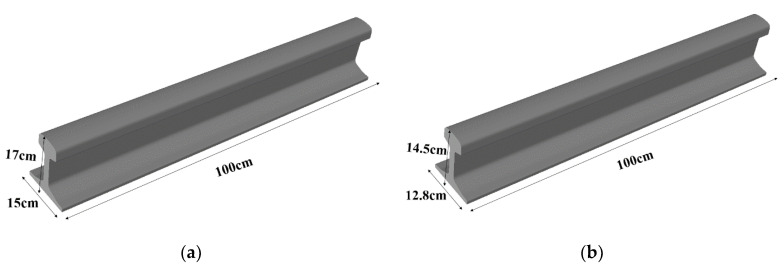
Schematic representations of test specimens: (**a**) 60 kg/m specimen; (**b**) 48 kg/m specimen.

**Figure 3:**
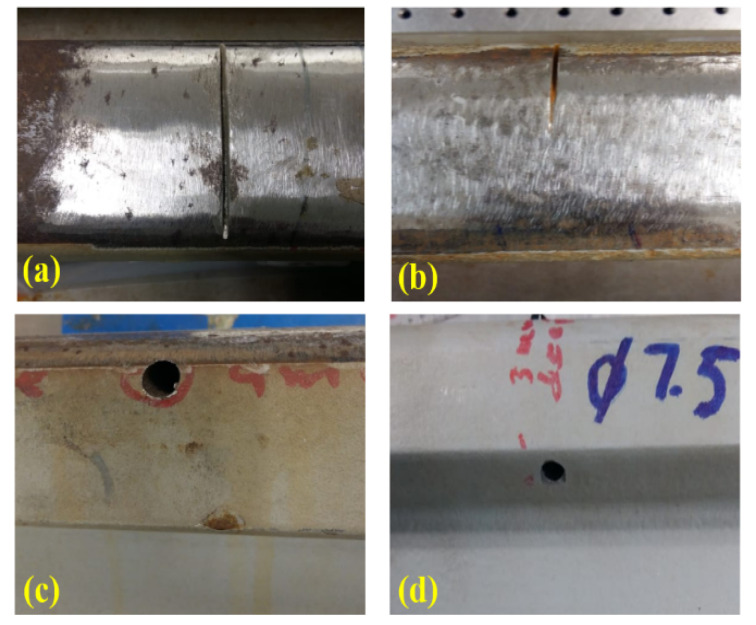
Real test specimens: (**a**) specimen B with surface horizontal defect; (**b**) specimen C with surface edge defect; (**c**) specimen D with subsurface horizontal defect; (**d**) specimen E with subsurface vertical defect.

In this study, five rail track specimens (A–E) were used. Hong Kong MTR Corporation Limited provided all the specimens. Among the test specimens, four (specimens A–B and D–E) were 60 kg in weight, while the fifth (specimen C) was smaller in size with a 48 kg weight. The dimensions of the specimens are shown in Figure 2. These specimens were rusted heavily at the railhead top surface, which could hinder wave propagation. To obtain smooth propagation of Rayleigh waves, the rust was removed with the help of a grinder machine. Table 1 presents the material properties of rail specimens. Specimen A was kept healthy, while artificial surface horizontal (3 mm deep, 2 mm wide and around 5.5 cm long) and edge (3 mm deep, 1.5 mm wide) defects were created in specimens B and C, respectively, as shown in Figure 3a,b. Similarly, artificial subsurface horizontal (4 mm deep, 7.5 mm in diameter) and vertical (3 mm deep, 7.5 mm in diameter) defects were created in specimens D and E, respectively, as shown in Figure 3c,d.

### 3.2. Experimental Setup

**Figure 4:**
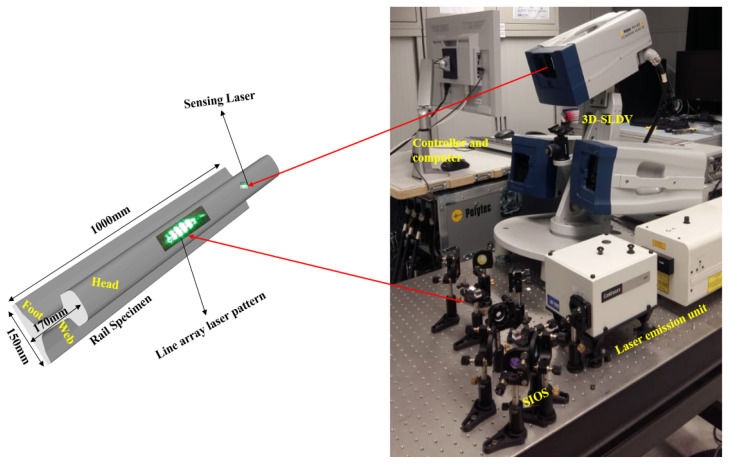
Schematic illustration of the experimental setup for non-contact inspection.

For experimental dictionary design and inspection of railheads, the schematic representation of the completely non-contact experimental setup is shown in Figure 4. A high-power Q-switched and pulsed laser, Nd: YAG, was used as an excitation source to emit a laser beam of 532 nm wavelength and 8 ns pulse duration. The emitted laser beam was passed through the lens setup called SIOS, which creates a line array pattern (LAP) of the incident laser beam. As a result of thermoelastic phenomena, this LAP generates Rayleigh waves in a narrowband frequency range. Further, the frequency of the generated Rayleigh waves was controlled by adjusting the LAP line width with mirror 3 (M3) [36].

A 3D SLDV (Polytec, PSV-500-3D-M, Polytech GmbH, Polytec-Platz 1-7 76337 Waldbronn, Germany) was used to sense the generated Rayleigh waves. The recorded signals were then transferred to a PC for signal processing by using enhanced matching pursuit. The 3D SLDV comprises three scanning heads, a junction box and a computer. It uses the Doppler shift phenomenon to measure surface motion in the form of the velocity of surface vibrations.

### 3.3. Properties of Laser-Generated Narrowband Ultrasound

**Figure 5:**
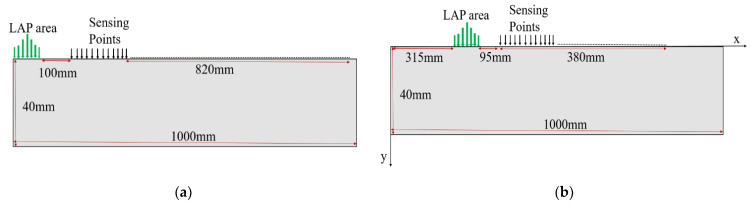
Schematic representation of experimental arrangements on healthy specimen A: (**a**) for wave propagation study and (**b**) to design dictionaries.

In order to study the properties of the generated narrowband ultrasonic waves at railheads by LAP laser excitation, a line scan was performed on a healthy rail specimen, A, as shown in Figure 5a. Depending on the depth and size of defects to be inspected (shown in Figure 3a–d, ultrasonic waves of around 400 kHz frequencies were generated. To obtain a clear signal, 170 measurements were averaged at each sensing point. The sampling frequency of the 3D SLDV was set to 5.12 MHz; further, at each measurement point, 8192 samples were recorded. To avoid near-surface effects, scanning started 10 cm away from the excitation area. The generated waves were recorded at 421 points with a spacing of 1.995 mm along the railhead’s centerline.

**Figure 6:**
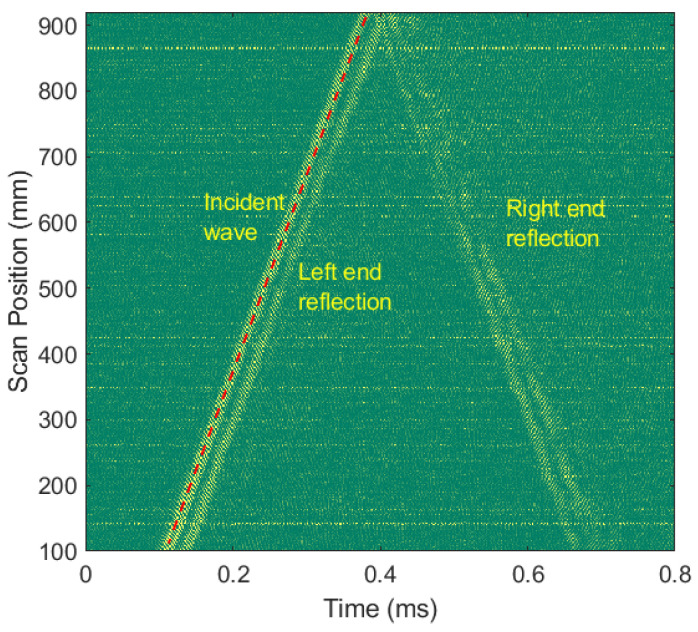
B-scan results using LAP laser excitation on the healthy railhead specimen A.

Figure 6 shows the B-scan of the envelope of the measured signals. Along with the incident wave, reflections from both ends of the rail are also observed. The left-end reflection is very close to the incident wave because the excitation laser pattern was kept close to the left end. The right-end reflection looks very complicated due to scattering and mode conversion at this rail end. Further, it consists of multimode interfering signals due to which the amplitude differs along the rail. However, despite the presence of multimode signals, the incident wave is quite a clearly defined wave packet. This incident wave velocity is 3046 m/s, which is close to the theoretical Rayleigh wave velocity (3055 m/s) in the steel railhead. The results have the same trend as that observed by Y. Fan et al. [40] while studying surface wave propagation on a railhead excited by EMAT, and they concluded that surface waves on the railhead could be treated as Rayleigh waves.

**Figure 7:**
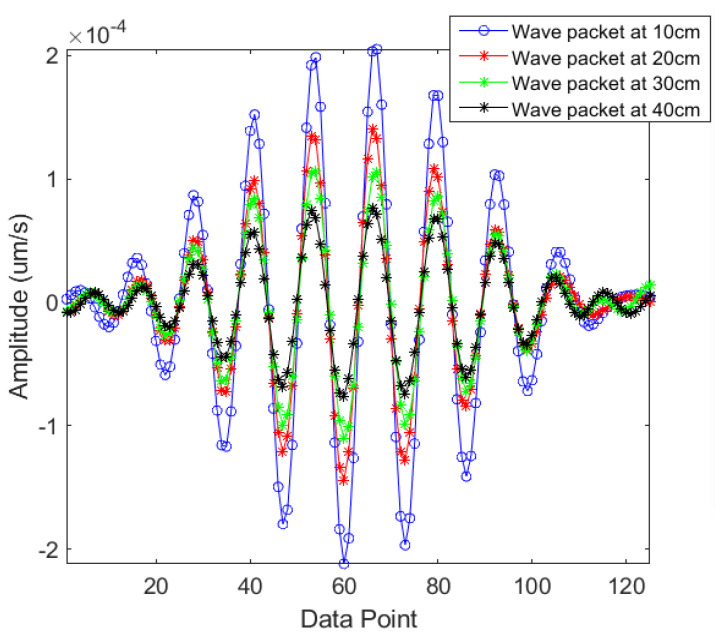
Wave packets of the signals measured at 10 cm, 20 cm, 30 cm and 40cm away from the excitation area.

Further, to check the dispersion in a laser-generated Rayleigh-like wave, its waveform was analyzed along the rail at different locations. The signals were measured at 10 cm, 20 cm, 30 cm and 40 cm away from the laser excitation area, and they were offset by an appropriate time to analyze their wave packets; the results are presented in Figure 7. It is observed that the wave packet of each signal is non-dispersive, and no noticeable distortion can be seen. Further, like a Rayleigh wave, the amplitude of the wave packets decreases gradually as the distance from the laser excitation area increases. Based on these experimental findings, the surface waves generated on railheads were considered as non-dispersive Rayleigh waves traveling with a reasonably consistent amplitude and frequency.

### 3.4. The Design of the Experimental Dictionary

To collect experimental signals for dictionary design, a line scan on specimen A was repeated with a new arrangement of excitation and detection lasers. The positions of excitation lasers and scanning points were arranged in a way that the end reflections do not appear in the required time window. The generated waves were recorded at 227 points with a spacing of 1.67 mm along the centerline of the railhead, as shown in Figure 5b.

**Figure 8:**
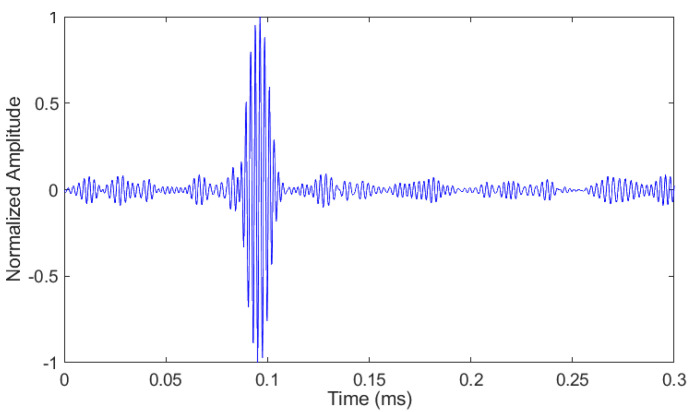
Time plot of an arbitrary atom from the experimental dictionary.

The experimental dictionary consisted of 227 atoms since all the recoded signals were added to it. For each atom, the sampling frequency and the number of samples were 5.21 MHz and 2048, respectively. The time plot of an atom from the dictionary is given in Figure 8. At 96.4 µs, this atom has a clear incident wave packet consisting of eight cycles. However, some unwanted wave packets and noise are also present that cannot be prevented in a real experimental measurement. The velocity of the incident wave packet is 3045 m/s, which shows that it is a Rayleigh wave, and, further, its frequency is in the narrowband with a central frequency of around 400 kHz.

## 4. Development of FEM Simulation Dictionary

A finite element method (FEM) simulation model using commercially available FEM software ABAQUS was developed to design an FEM simulation dictionary. It has been reported [41,42] that using a 2D model with plane strain approximation can successfully predict the propagation and interaction of ultrasonic waves with defects in railheads. Further, the aim was to develop a simulation dictionary whose constituent atoms have similar characteristics to those of experimentally generated Rayleigh waves. Hence, to save computational time, the FEM simulation dictionary was designed by developing a 2D plane type of FEM simulation. The background of the thermoelastic generation of ultrasonic waves and the simulation model’s details are described in the subsequent subheadings.

### 4.1. Thermoelastic Theory

When a pulse laser irradiates a sample surface, it absorbs energy, and a transient temperature field is generated, which induces a stress field in the material (thermoelastic phenomenon). The resulting thermoelastic coupling control equation can be expressed as:

(6) $$\rho C_{p}\frac{\partial T\left(x,y,t\right)}{\partial t}=▽\left(k▽T\left(x,y,t\right)\right)+Q$$

*T*(*x*, *y*, *t*) shows the temperature distributions with respect to time along the *x* and *y* directions. *Q*, *k*, *Cp* and *ρ* represent heat source, thermal conductivity, specific heat capacity at constant pressure and density, respectively. Since, instead of a heat source, a pulse laser is usually treated as heat flux, *Q* = 0.

The normal boundary conditions of the specimen by considering the skin effect of the steel solid are given by:

(7) $$-k\frac{\partial T\left(x,y,t\right)}{\partial y}{|}_{y=0}=If\left(x,y\right)g\left(t\right)$$

(8) $$\frac{\partial T\left(x,y,t\right)}{\partial y}{|}_{y=h}=0$$

where *g* (*t*) and *f*(*x*, *y*) are temporal and spatial distributions of the excitation laser, *I* is its energy density and *h* is the railhead thickness. In this study, the spatial distribution *f(x*, *y)* has eight laser lines (interference fringes) with spacing equal to the wavelength (*λ*) of the produced ultrasonic waves. By taking *f*(*x*, *y*) as a Gaussian distribution function, the intensity distribution of the line array laser pattern formed by the laser beam is given by [36].

(9) $$f_{n}\left(x,y\right)=\frac{1}{\sigma \sqrt{2\pi }}e^{-\frac{x^{2}+y^{2}}{2\sigma^{2}}cos^{2}\left(\frac{\pi }{d}x\right)}$$

where *n* is the number of lines in the LAP, *x* and *y* are coordinates of the laser illumination region, *σ* is 1/6 of the incident laser beam’s diameter and *d* is the distance between two laser lines.

Similarly, the temporal distribution of the laser is also approximated as a Gaussian distribution function and is given by [15].

(10) $$g\left(t\right)=\frac{8t^{3}}{t_{0}^{4}}\exp\left(-\frac{2t^{2}}{t_{0}^{2}}\right)$$

where *t*_0_ is the rising time of the laser.

### 4.2. Simulation Model

For the dictionary design, like the original railhead, a 2D plane model with 40 × 1000 mm dimensions was developed, as shown in Figure 5b. The x-axis denotes the path of wave propagation along the railhead, and the y-axis shows its thickness. In order to avoid end reflections, absorbing boundaries were applied at the left and right sides of the model. Further, since, in this study, Rayleigh waves were mainly used for detect detection, and they can penetrate only a few wavelengths of the incident wave, absorbing boundaries were also applied to the bottom of the models. Table 1 presents the material properties of the steel rail.

A pulse laser as a heat flux was applied onto the railhead’s top surface at x = 315, y = 0. The initial temperature of the models and environments was kept at 300 K. The model parameters are given in Table 2.

**Figure 9:**
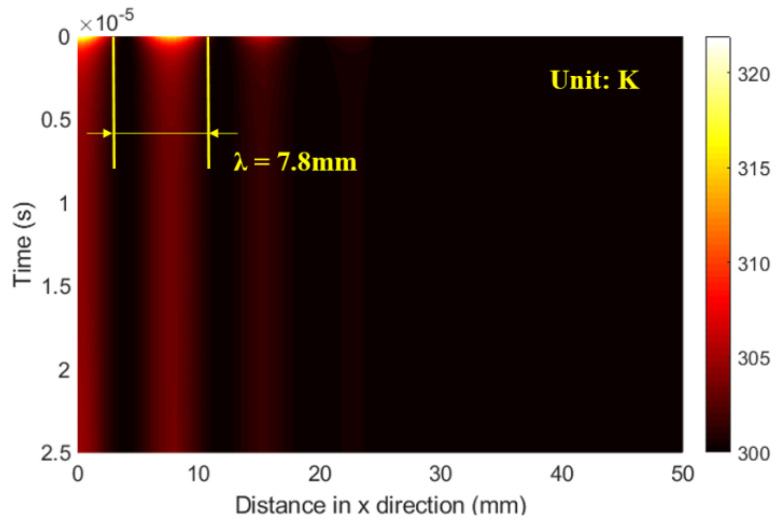
Temperature field distribution as a result of thermoelastic laser excitation when λ = 7.8 mm.

By taking λ = 7.8 mm, the distribution of time histories of the temperature field taken at the railhead surface is given in Figure 9. Here, half of the temperature field was simulated because it is symmetric at the laser irradiance location. It is observed that the temperature increase is limited to the line array region of the railhead, where the laser heat flux was irradiated. The temperature is highest at the bright central fringe, and then it decreases toward the first and second bright fringes. This shows that the temperature is limited to the irradiance area, and far from this area, no temperature change is evidenced.

### 4.3. The Design of the FEM Simulation Dictionary

**Figure 10:**
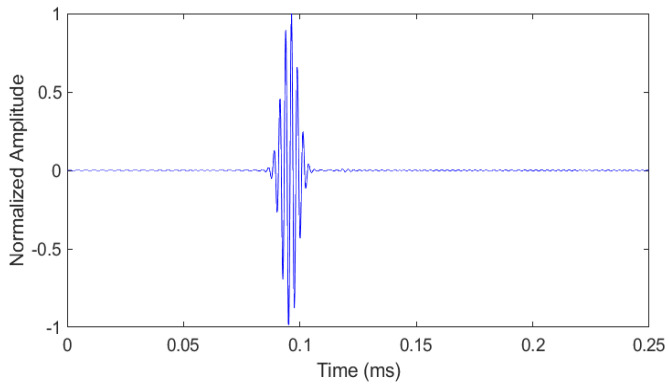
Time plot of an arbitrary atom selected from the FEM simulation dictionary.

The time plot of an arbitrary atom selected from the dictionary is given in Figure 10. Here, a clear incident wave at 96.4 µs can be observed from the graph. It is observed that the waveform of this atom is quite similar to the experimental atom (Figure 8) as both have the same number of lines. Further, the velocity and frequency of the simulated wave are 3040 m/s and 400 kHz, respectively. These velocity and frequency values are close to those of the experimental atom. This shows that the proposed simulation model successfully generated a Rayleigh wave having the same characteristics as an experimentally generated one. From the modal total, 227 signals were recorded with 1.67 mm spacing. Therefore, the FEM simulation dictionary consisted of 227 atoms.

## 5. Results and Discussion

### 5.1. Defect Detection by the Non-Contact Laser-Based Inspection System

**Figure 11:**
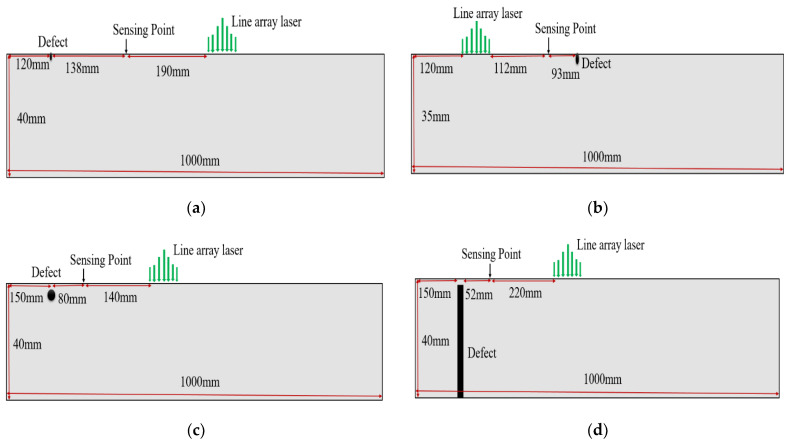
Schematic representation of the experimental arrangement for defect detection in railheads: (**a**) specimen B with surface horizontal defect, (**b**) specimen C with surface edge defect, (**c**) specimen D with subsurface horizontal defect and (**d**) specimen E with subsurface vertical defect.

The effective non-contact generation and sensing of narrowband Rayleigh waves and their ability to detect surface and subsurface defects in railheads were investigated by the completely non-contact laser-based inspection system described in Section 3.2. The laser excitation locations and receiving points for all defective specimens, B, C, D and E, are given in Figure 11a–d, respectively. The position of the LAP center was kept in a way that end reflections do not appear in the given time window. Depending on the depth and size of defects, Rayleigh waves of around 400k Hz frequencies were generated for all surface and subsurface defects.

**Figure 12:**
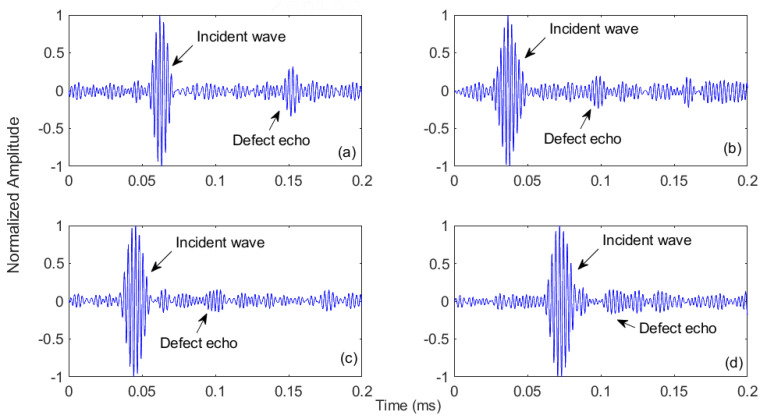
Time plots of the captured signals: time plots of (**a**) specimen B with surface horizontal defect, (**b**) specimen C with surface edge defect, (**c**) specimen D with subsurface horizontal defect and (**d**) specimen E with subsurface vertical defect.

For rail specimen B, with a surface horizontal defect, the propagation of waves along the out-of-plane direction was captured at the sensing point located 13.8 cm away from the defect. Figure 12a shows the time plot of the captured signal. The graph shows that the incident Rayleigh wave arrives at the sensing point at 62.2 µs, followed by a strong defect echo at 153 µs. The time plot of the signal recorded at the sensing point of specimen C is given in Figure 12b. A prominent incident wave packet is evidenced at 36.9 µs; however, the reflection echo that appeared at 97.5 µs is relatively weak and further noisy wave packets surround it. This is because the edge defect was at the curved surface of the small rail. For specimen D, with a subsurface horizontal defect, the temporal plot is given in Figure 12c. Here, the incident wave appears at 45.8 µs. However, the defect echo is not clear because components having almost the same amplitude are surrounding it. Based on the estimated arrival time of the defect echo, the defect reflection location is indicated at 98.2 µs. Similarly, the defect echo captured at 106.2 µs from the subsurface vertical defect is also surrounded by unwanted wave packets, although the incident wave is very clear, as shown in Figure 12d. These time plots of the captured signals at all defective rail specimens (B–E) show that laser-generated excitations are usually contaminated with noise. Therefore, generally, signal processing techniques are required to extract defect echoes from a laser-generated ultrasonic signal.

### 5.2. Demonstration of Proposed MP for Defect Detection

**Figure 13:**
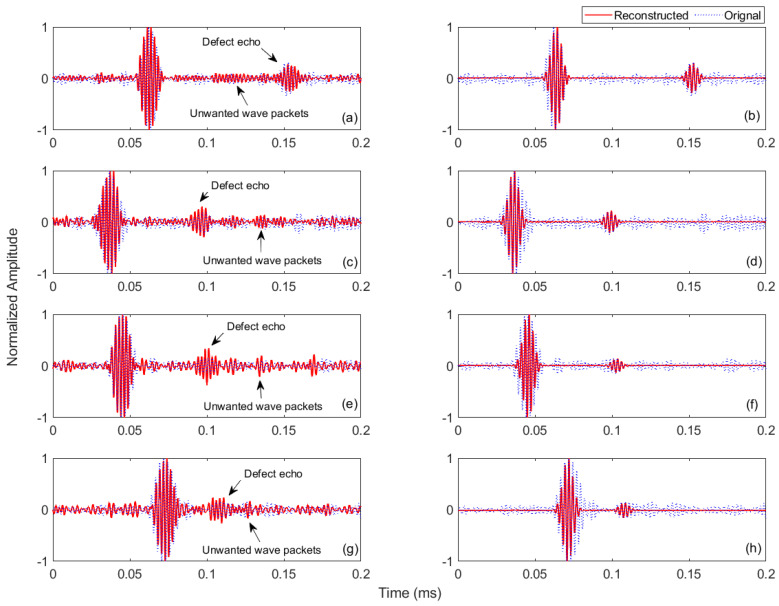
Original captured signals and their reconstructed signals after MP with the experimental dictionary: (**a**) specimen B with surface horizontal defect, (**c**) specimen C with surface edge defect, (**e**) specimen D with subsurface horizontal defect and (**g**) specimen E with subsurface vertical defect; and after MP with the FEM simulation dictionary: (**b**) specimen B with surface horizontal defect, (**d**) specimen C with surface edge defect, (**f**) specimen D with subsurface horizontal defect and (**h**) specimen E with subsurface vertical defect.

As described earlier, the dictionary atoms are designed to match the significant ultrasonic echoes maximally and mismatch the correlated noise. In this regard, MP, along with the designed dictionaries consisting of FEM simulation and real experimental laser-generated signals, was applied separately to experimental signals to locate the defect echoes and remove unwanted components. For the enhanced MP algorithm, the value of the residual norm was kept as 0.1 for both experimental and simulation dictionaries. The reconstructed signals, after MP with the experimental dictionary, measured at the sensing points of specimens B, C, D and E are shown in Figure 13a,c,e,g, respectively. It is observed that MP with the experimental dictionary successfully approximates the incident wave and defect echoes in all signals, although in most of the signals, defect echoes were heavily surrounded by unwanted wave packets, as shown in Figure 12a–d. The original waveforms of the incident wave and defect echoes are well captured, and notably, amplitudes of the defect echoes are also very strong. However, the reconstructed signals still contain some noise but of relatively low amplitude, as indicated in Figure 13a,c,e,g, where some prominent unwanted wave packets are highlighted. This is because, in practice, experimental signals are usually contaminated by noise associated with the measurement system, test specimen and environment. Due to this, all the atoms in the dictionary contained some noise, and consequently, the reconstructed signal cannot be noise-free. Here, the significant finding is that the experimental dictionary was designed from signals captured at a healthy railhead and they successfully match (through MP) with signals containing defect echoes (from different surface and subsurface defects) measured at rails of different dimensions (48 Kg/m and 60 Kg/m). This shows the potential widespread applicability of enhanced MP with the experimental dictionary in the rail industry where rails of various sizes are being used.

**Figure 14:**
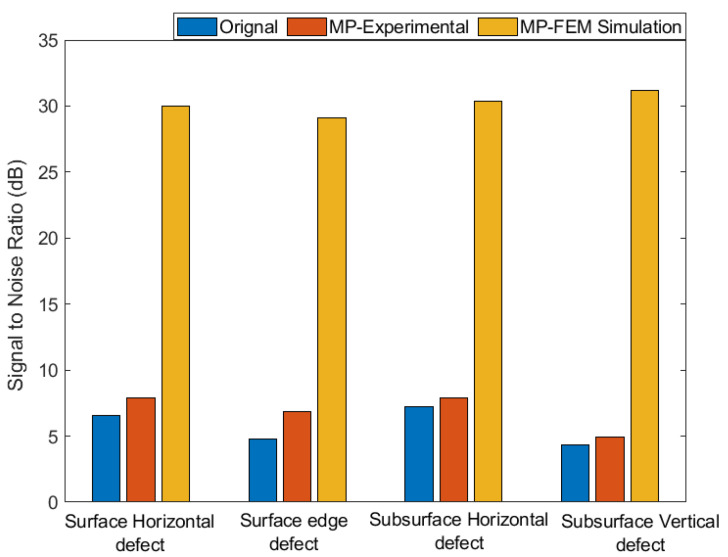
Comparison of SNR values of the original signals and reconstructed signals after MP with the experimental and FEM simulation dictionaries for specimen B with surface horizontal defect, specimen C with surface edge defect, specimen D with subsurface horizontal defect and specimen E with subsurface vertical defect.

The reconstructed signals after MP with the simulation dictionary are shown in Figure 13b,d,f,h for specimens B, C, D and E, respectively. Here, the reconstructed signals contain only the incident wave and defect echoes, and all other unwanted components disappear. However, in these reconstructed signals, the original waveforms of the incident wave and defect echoes are not well captured. But, this mismatch in waveforms will not significantly affect the defect detection accuracy, as described in the next paragraph. A comparison of both experimental and simulation dictionaries to suppress noise is presented in Figure 14, where the signal-to-noise ratios (SNRs) of the original signals and reconstructed signals after MP with experimental and simulation dictionaries are given. From Figure 14, it is observed that compared to the original signals, the SNRs of the reconstructed signals after MP with the experimental dictionary improved slightly, which are from 6.5 to 7.9, 4.8 to 6.8, 7.2 to 7.8 and 4.3 to 4.9 for the surface horizontal defect, surface edge defect, subsurface horizontal defect and subsurface vertical defect, respectively. On the other hand, compared to the original signals, the SNRs of the reconstructed signals after MP with the FEM simulation dictionary improved significantly, from 6.5 to 30.0, 4.8 to 29.1, 7.2 to 30.4 and 4.3 to 31.2 for the surface horizontal defect, surface edge defect, subsurface horizontal defect and subsurface vertical defect, respectively. These results show that MP with the simulation dictionary provides excellent results in terms of removing noise and unwanted wave packets.

Next, the location of each defect was measured by using reconstructed signals through enhanced MP. Since the enhanced MP with both experimental and simulation dictionaries successfully approximated the incident wave and defect echoes, the pulse-echo method was used to measure the defect location. The comparison of both dictionaries in locating defects is presented in Table 3. It is observed that surface horizontal, surface edge, subsurface horizontal and subsurface vertical defect location (DL) errors for MP with the experimental dictionary are 1.78%, 2.26%, 1.85% and 2.57%, respectively. These values are less than those for MP with the simulation dictionary that are 2.30%, 2.80%, 3.36% and 3.48% defect location errors for surface horizontal, surface edge, subsurface horizontal and subsurface vertical defects, respectively. The relatively higher accuracy of defect detection with the experimental dictionary is attributed to its ability to fully capture the original waveforms of the incident and defect echoes. Although the defect location errors of MP with the FEM simulation dictionary are higher than those from MP with the experimental dictionary, this difference is very small and can be acceptable in industrial practices.

Note that although the atoms in the FEM simulation dictionary were generated by designing a 2D model with plane strain approximation, the defect detection accuracy of the enhanced MP with the FEM simulation dictionary is very close to that of the MP with the experimental dictionary. This means that the experimental dictionary validates the reliability of the FEM simulation dictionary. Therefore, the proposed FEM simulation dictionary is almost as equally reliable as an experimental dictionary and can save high equipment costs and long experiment times for the generation of real laser ultrasonic signals for the development of an experimental dictionary. Hence, the enhanced MP with the FEM simulation dictionary can be effectively used for noise suppression and defect detection from a laser-generated Rayleigh wave signal captured at the railhead.

### 5.3. Demonstration of Enhanced MP to Overcome Low Repeatability of Laser-Generated Rayleigh Wave Signals

**Figure 15:**
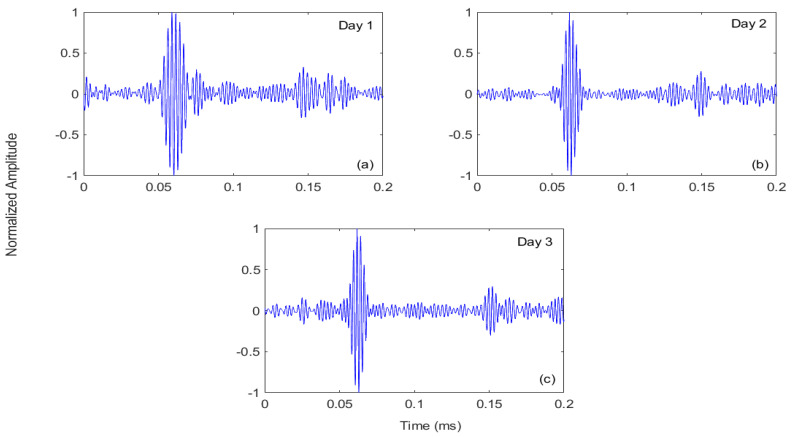
The signals captured on consecutive days: (**a**) day 1, (**b**) day 2 and (**c**) day 3, at specimen B that has a surface horizontal defect.

One of the significant drawbacks of laser excitation on railheads is that the measurements are usually non-repeatable. It is observed that the shape of the incident wave and the appearance of noise look different at the same detection point when time-domain signal measurements are taken at other times. The changing waveforms over time of the measured signals make the applicability of signal processing techniques very difficult, particularly when laser-generated signals are highly contaminated by noise and unwanted wave packets. To investigate the low repeatability of laser-generated Rayleigh signals, experiments were repeated for three consecutive days with the same excitation and sensing location of sample B with a surface horizontal defect (Figure 11a), and the captured signals are shown in Figure 15a–c. It is observed that the incident wave packet slightly changes in all three signals; however, the variation in noise level and unwanted wave packets is quite noticeable. The day 1 signal is more contaminated with unwanted wave packets; however, from day 2 to day 3 measurements, the number and amplitude of surplus wave packets decrease.

**Figure 16:**
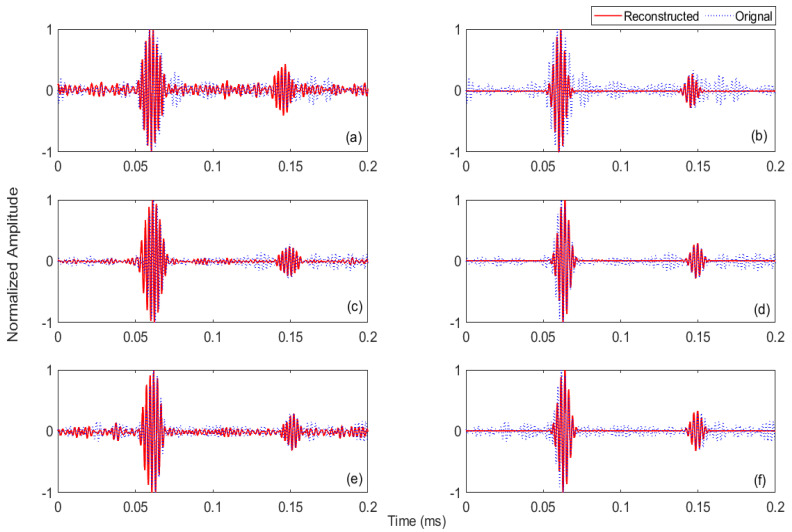
Original day-wise signals captured at specimen B that has a surface horizontal defect and their reconstructed signals after MP with the experimental dictionary: (**a**) day 1, (**c**) day 2 and (**e**) day 3; and after MP with the FEM simulation dictionary: (**b**) day 1, (**d**) day 2 and (**f**) day 3.

The robustness of the enhanced MP with FEM simulation and experimental dictionaries to overcome the low repeatability of laser-generated Rayleigh signals was checked for the recorded signals (in Figure 15a–c). First, MP with the experimental dictionary was applied to these measured signals. The reconstructed signals after MP are shown in Figure 16a,c,e for day 1, day 2 and day 3, respectively. These results show that MP with the experimental dictionary successfully extracts the incident waves and defect echoes, although the measured signals had different noise levels and unwanted wave packets. Further, waveforms of the incident and defect echoes are well captured. Next, MP with the FEM simulation dictionary was applied to the signals, and the results are presented in Figure 16b,d,f for day 1, day 2 and day 3, respectively. It is observed that after using MP with the FEM simulation dictionary, in all signals, only incident Rayleigh waves and defect deflections are present, while all other unwanted compounds disappear. However, there is some mismatch between the waveform of the actual and reconstructed incident waves that may affect the accuracy of the defect location measurement.

The comparison of both dictionaries in locating defects from day-wise signals is presented in Table 4. The day-wise defect location error for MP with the experimental dictionary is 3.42%, 2.33% and 0.80% for day-1, day-2 and day-3 readings, respectively. These defect location errors are slightly less than those for MP with the simulation dictionary that are 4.90%, 3.73% and 1.52% for day-1, day-2 and day-3 readings. Hence, the enhanced MP with both experimental and FEM simulation dictionaries can be beneficial in overcoming variation in the captured laser-based reflection signals. However, in terms of suppressing noise and unwanted wave components, MP with the simulation dictionary is still the primary option.

## 6. Conclusions

This study explored the possibilities of combining laser ultrasonic technology and an enhanced MP to support a fully non-contact inspection of railheads. It is well known that non-contact laser-generated ultrasonic signals propagating along steel specimens usually have a lower signal-to-noise ratio (SNR). To minimize noise and unwanted wave packets, an enhanced MP, along with newly designed FEM simulation and experimental dictionaries, was proposed to assist the laser-generated Rayleigh wave signals in the process of defect detection. The capabilities of the enhanced MP were investigated for signals obtained from defective rail specimens B, C, D and E having surface horizontal, surface edge, subsurface horizontal and subsurface vertical defects, respectively. These signals were generated by a fully non-contact laser-based inspection system. MP with the experimental dictionary successfully located the incident wave and defect echo and suppressed the unwanted wave packets to some extent, apart from some unavoidable noise generated in real experiments. The original waveform of the measured signals was well approximated that helped in the accurate measurement of the defect location.

On the other hand, MP with the FEM simulation dictionary was also found to be highly efficient in suppressing noise and unwanted wave packets. However, its defect location measurement accuracy was relatively smaller than MP with the experimental dictionary, but this difference was minimal and can be acceptable in industrial practices. Notably, this small difference in defect location measurement accuracies between experimental and FEM simulation dictionaries also shows that the experimental dictionary validated the reliability of the FEM simulation dictionary. Further, the enhanced MP with the FEM simulation dictionary was also proved to be very beneficial in overcoming the low repeatability of laser-generated signals. Therefore, this research concludes that the proposed enhanced MP with the FEM simulation dictionary can effectively assist in the non-contact defect detection of a railhead by de-noising laser-generated Rayleigh wave signals captured on its surface.

Future studies will be continued and will emphasize developing an MP with a hybrid dictionary that contains the advantages contributed by both dictionaries so that the effect of suppressing noise and the enhanced ability in locating incident and defect echoes can be obtained simultaneously. Equipped with such MP, more efficient noise-free defect detection of laser-generated ultrasonic signals could be realized in rails.

## Figures and Tables

**Table 1:** Material properties of the steel rail specimen.

Young’s Modulus *E* (GPa)	Poisson’s Ratio *σ*	Density *ρ* (kg/m^3^)	Specific Heat (J/KgK)	Conductivity (W/mK)	Rayleigh Velocity (m/s)
220	0.3	7800	475	44.5	3055

**Table 2:** Simulation model parameters.

λ (d)	σ	Time Step (Thermal/Elastic)	Mesh Size (Thermal/Elastic)	Total Time (Thermal/Elastic)
7.8	10	1.5/180 ns	0.03/0.38 mm	25/400 µs

**Table 3:** Comparison of MP with FEM simulation and experimental dictionaries to locate railhead surface and subsurface defects.

Defect Type	DL_ Original Waveform (cm)	DL_ MP with Simulation Dictionary (cm)	DL_ MP with the Experimental Dictionary (cm)	Error_ MP with Simulation Dictionary (%)	Error_ MP with the Experimental Dictionary (%)
Surface Horizontal	13.8	13.482	13.555	2.30	1.78
Surface Edge	9.3	9.56	9.09	2.80	2.26
Subsurface Horizontal	8.0	8.27	8.15	3.36	1.85
Subsurface Vertical	5.2	5.381	5.066	3.48	2.57

**Table 4:** Comparison of MP with FEM simulation and experimental dictionaries to locate defects from day 1 to day 3 measurements.

	DL_ Original Waveform (mm)	DL_ MP with Simulation Dictionary (mm)	DL_ MP with the Experimental Dictionary (mm)	Error_ MP with Simulation Dictionary (%)	Error_ MP with the Experimental Dictionary (%)
Day 1	13.8	13.124	13.328	4.90	3.42
Day 2	13.8	13.285	13.479	3.73	2.33
Day 3	13.8	13.590	13.690	1.52	0.80
